# *Kamishoyosan* and *Kamikihito* protect against decreased KCC2 expression induced by the *P. gingivalis* lipopolysaccharide treatment in PC-12 cells and improve behavioral abnormalities in male mice

**DOI:** 10.1016/j.heliyon.2023.e22784

**Published:** 2023-11-25

**Authors:** Kazuo Tomita, Yukiko Oohara, Kento Igarashi, Junichi Kitanaka, Nobue Kitanaka, Koh-ichi Tanaka, Mehryar Habibi Roudkenar, Amaneh Mohammadi Roushandeh, Mitsutaka Sugimura, Tomoaki Sato

**Affiliations:** a Department of Applied Pharmacology, Kagoshima University Graduate School of Medical and Dental Sciences, Kagoshima, 890-8544, Japan; b Division of Pharmacology, Department of Pharmacy, School of Pharmacy, Hyogo Medical University, Hyogo, 650-8530, Japan; c Department of Dental Anesthesiology, Kagoshima University Graduate School of Medical and Dental Sciences, Kagoshima, 890-8544, Japan; d Laboratory of Drug Addiction and Experimental Therapeutics, School of Pharmacy, Hyogo Medical University, Hyogo, 650-8530, Japan; e Department of Pharmacology, School of Medicine, Hyogo Medical University, Hyogo, 663-8501, Japan; f Burn and Regenerative Medicine Research Center, Velayat Hospital, School of Medicine, Guilan University of Medical Sciences, Rasht, 41937-13194, Iran; g Department of Anatomy, School of Biomedical Sciences, Medicine & Health, UNSW Sydney, Sydney, NSW, 2052, Australia

**Keywords:** KCC2, LPS, GABA, Oxytocin, PC-12 cells

## Abstract

*Kamishoyosan* (KSS) and *Kamikihito* (KKT) have been traditionally prescribed for neuropsychiatric symptoms in Japan. However, the molecular mechanism of its effect is not elucidated enough. On the other hand, it has been reported that lipopolysaccharide derived from *Porphyromonas gingivalis* (*P. g* LPS) is involved not only in periodontal disease but also in the systemic diseases such as psychiatric disorders via neuroinflammation. Here, we investigated the molecular mechanism of KSS and KKT treatment by LPS-induced neuropathy using PC-12 cells. When *P. g* LPS was administrated during the NGF treatment, the KCC2 expression was decreased in PC-12 cells. *P. g* LPS treatment also decreased the WNK and phospho SPAK (pSPAK) expression and enhanced GSK-3β expression that negatively regulates WNK-SPAK signaling. Moreover, when KSS or KKT was administrated before *P. g* LPS treatment, the decrease of KCC2, WNK and pSPAK was rescued. KSS and KKT treatment also rescued the enhancement of GSK3β expression by *P. g* LPS treatment. Furthermore, KSS, KKT and/or oxytocin could rescue behavioral abnormalities caused by *P. g* LPS treatment by animal experiments. These effects were not shown in the Goreisan treatment, which has been reported to act on the central nervous system. These results indicate that KSS and KKT are candidates for therapeutic agents for neural dysfunction.

## 1 Introduction

Japanese herbal medicines (Kampo) such as *Kamishoyosan* (KSS) and *Kamikihito* (KKT) are prescribed for neuropsychiatric symptoms in Japan. For example, KSS is prescribed to male patients with autonomic dysfunction to improve fatigue and palpitations [1]. KKT is prescribed to patients with anxiety, depression, and insomnia [2]. A recent report demonstrated that KSS reduces aggressive biting behavior, which is an indicator of irritability, through the regulation of serotonin (5-hydroxytryptamine; 5-HT) expression and estrogen receptors [3]. KKT may also increase the concentration of oxytocin (OXT) in cerebrospinal fluid [4], leading to OXT-mediated stress reduction in the nervous system.

OXT is a neuropeptide synthesized in the hypothalamus and acts as a neurotransmitter in the central nervous system [5,6]. OXT may be involved in the regulation of peripheral and central nervous functions, including social behavior [5]. It has been reported that autism symptoms improved in clinical trials in which OXT was administered through the nasal mucosa [7,8]. OXT is also involved in anti-inflammation [9], glycogen synthase kinase-3β (GSK-3β) signaling [10], maintenance of plasma membrane potential [11], and the GABA switch by up-regulating K^+^-Cl^-^ co-transporter 2 (KCC2) expression [12].

Lipopolysaccharides (LPS) derived from the periodontal pathogen *Porphyromonas gingivalis* (*P. g* LPS) induce inflammation and act as a stressor involved in the development of periodontal disease and systemic diseases, such as neuropsychiatric symptoms [[13], [14], [15]]. It has been reported that *P. g* LPS induce neuroinflammation by up-regulating IL-1β [11]. LPS derived from *Escherichia coli* causes depression-like behavior [16] and downregulates KCC2 in mice [17]. *P. g* LPS treatment decreases the expression of KCC2, which is important for the maturation of the central nervous system. OXT treatment can attenuate or prevent the decreased KCC2 expression induced by *P. g* LPS [11]. Therefore, Kampo such as KKT may prevent the decreased KCC2 expression induced by LPS via the stress-protective effects of OXT. However, the relationships between the Kampo and KCC2 expression are not clear.

The mechanism by which LPS decreases KCC2 expression has been partially elucidated. LPS binding to the cell surface receptor toll-like receptor (TLR) 4 stimulates macrophages to release inflammatory cytokines, such as interleukin-1 beta (IL-1β). IL-1β binds to the IL-1R and promotes nuclear translocation of RE1-silencing transcription factor (REST) and methyl CpG binding protein 2 (MECP2), which bind to the transcriptional regulatory region of KCC2 and downregulate expression [18]. Moreover, GSK3β is involved in the LPS-induced downregulation of KCC2 expression, and OXT rescues the *P. g* LPS-induced downregulation of KCC2 expression via GSK3β [11]. GSK3β affects mitochondrial activity, causing a decrease in mitochondrial membrane potential (Ψ_m_) and the generation of mitochondrial reactive oxygen species (mtROS) [19]. GSK3β also acts as a positive effector downstream of the with-no-lysine kinase (WNK)-STE20/SPS1-related proline/alanine-rich kinase (SPAK) system [20]. The WNK-SPAK system regulates the expression of KCC2 and Na^+^-K^+^-Cl^-^ co-transporter 1 (NKCC1) in renal cells by phosphorylating Ser373 of SPK [21,22]. Moreover, GSK3β is activated by the receptor for advanced glycation end products (RAGE), which acts as a receptor for LPS and OXT [23,24]. However, the involvement of the WNK-SPAK system and maintenance of mitochondrial function in regulating the expression of KCC2 expression during the maturation of the central nervous system is unclear.

Therefore, we investigated the involvement of the WNK-SPAK system and mitochondria in the regulation of KCC2 expression during neural maturation using PC-12 cells. We also investigated the effect of Kampo on *P. g* LPS-induced downregulation of KCC2 expression. Furthermore, we conducted animal experiments to investigate whether Kampo and/or OXT could rescue behavioral abnormalities caused by *P. g* LPS treatment.

## 2 Materials and methods

### 2.1 PC-12 cell culture and differentiation

PC-12 cells, which is derived from a pheochromocytoma of the rat adrenal medulla, were provided by the RIKEN BRC through the National BioResource Project of the MEXT/AMED, Japan. Cells were cultured in RPMI 1640 (189-02025: Fujifilm Wako, Osaka, Japan) with 5% horse serum (HS; 26050-088: Life Technologies NZ Ltd., Auckland, NZ), 5% fetal bovine serum (04-001-1A: Biological Industries, Cromwell, CT, USA), and 1% penicillin-streptomycin (PS). PC-12 cells were cultured in RPMI1640 with 100 ng/ml of nerve growth factor (NGF; N0513: Sigma-Aldrich, St Louis, MO, USA), 1% HS, and 1% PS for five days to induce differentiation for subsequent experiments.

### 2.2 Lipopolysaccharides, oxytocin, and Kampo treatment of PC-12 cells

*P. g* LPS (strain ATCC 33277; ppglps: InvivoGen, San Diego, CA, USA) was added to the medium at a concentration of 10 μg/ml. One hour after LPS treatment, the medium was replaced with differentiation media, and cells were cultured for five days without LPS. Cells were treated with 1 μM Cys-Tyr-Ile-Gln-Asn-Cys-Pro-Leu-Gly-NH2 (disulfide bond between Cys1-Cys6) (OXT) (4084-v: Peptide Institute, Osaka Japan) for 30 min before LPS treatment. KSS (TJ-24, Tsumura Co. Ltd., Tokyo, Japan; Serial No. F40842), KKT (TJ-137, Tsumura; Serial No. T05192), and *Goreisan* (GRS, TJ-17 Tsumura; Serial No. U21191) were purchased from Tsumura as a freeze-dried powder. The components and amounts of KSS, KKT, and GRS are listed in Table 1. Tsumura's herbal medicines are extracted according to the traditional method of traditional Chinese medicine, and the extracted extract is solid-liquid separated, then subjected to low-temperature, short-time concentration, and drying processes to produce extract powder. Preparation of the final formulation, including quality control data can be available from Tsumura. 3D-HPLC fingerprint of KSS, KKT, and GRS were provided by Tsumura Co, Ltd and shown in Supplemental Fig. 1. Tsumura Co, Ltd has confirmed that no specific LPS-producing microorganisms, *Escherichia coli* or Salmonella, have been detected in these herbal medicines. These Kampo were mixed and extracted with purified water at room temperature for 24 h. Cells were treated with 400 μg/ml KSS, 100 μg/ml KKT, or 400 μg/ml GRS for 30 min before LPS treatment. These concentrations were decided referring to the effective dosages in past reports [25,26].

**Table 1:** The component galenicals of kampo used in this study.

	*Kamishoyosan* (KSS)	
The component of galenicals	English name, Official name (botanical family)	Amount
Saiko	Bupleurum Root, Root of Bupleurum falcatum Linné (Umbelliferae)	3
Syakuyaku	Paony Root, Root of Paeonia lactiflora Pallas (Paeoniaceae)	3
Soujyutsu	Atractylodes Lancea Rhizome, Rhizome of Atractylodes lancea De Candolle (Compositae)	3
Touki	Japanese Angelica Root, Root of Angelica acutiloba Kitagawa (Umbelliferae)	3
Bukuryo	Poria Sclerotium, Sclerotia of Wolfiporia cocos Ryvarden et Gilbertson (Polyporaceae)	3
Sanshishi	Gardenia Fruit, Fruit of Gardenia jasminoides Ellis (Rubiaceae)	2
Botanpi	Moutan Bark, Root bark of Paeonia suffruticosa Andrews (Paeoniaceae)	2
Kanzo	Glycyrrhiza, Root of Glycyrrhiza glabra Linné (Leguminosae)	1.5
Shokyo	Ginger, Rhizome of Zingiber officinale Roscoe (Zingiberaceae)	1
Hakka	Mentha Herb, Ground part of Mentha arvensis Linné var. piperascens Malinvaud (Labiatae)	1
	*Kamikihito* (KKT)	
The component of galenicals	English name, Official name (botanical family)	Amount
Ougi	Astragalus Root, Root of Astragalus membranaceus Bunge (Leguminosae)	3
Saiko	Bupleurum Root, Root of Bupleurum falcatum Linné (Umbelliferae)	3
Sansounin	Jujube Seed, Seed of Ziziphus jujuba Miller var. spinosa Hu ex H. F. Chow (Rhamnaceae)	3
Soujyutsu	Atractylodes Lancea Rhizome, Rhizome of Atractylodes lancea De Candolle (Compositae)	3
Ninjin	Ginseng, Root of Panax ginseng C. A. Meyer (Araliaceae)	3
Bukuryo	Poria Sclerotium, Sclerotia of Wolfiporia cocos Ryvarden et Gilbertson (Polyporaceae)	3
Ryuganniku	Longan Aril, Arils of Euphoria longana Lamarck (Sapindaceae)	3
Onji	Polygala Root, Root of Polygala tenuifolia Willdenow (Polygalaceae)	2
Sanshishi	Gardenia Fruit, Fruit of Gardenia jasminoides Ellis (Rubiaceae)	2
Taisou	Jujube, Fruit of Ziziphus jujuba Miller var. inermis Rehder (Rhamnaceae)	2
Touki	Japanese Angelica Root, Root of Angelica acutiloba Kitagawa (Umbelliferae)	2
Kanzo	Glycyrrhiza, Root of Glycyrrhiza glabra Linné (Leguminosae)	1
Shokyo	Ginger, Rhizome of Zingiber officinale Roscoe (Zingiberaceae)	1
Mokko	Saussurea Root, Root of Saussurea lappa Clarke (Compositae)	1
	*Goreisan* (GRS)	
The component of galenicals	English name, Official name (botanical family)	Amount
Takusya	Alisma Tuber, Tuber of Alisma orientale Juzepczuk (Alismataceae)	4
Soujyutsu	Atractylodes Lancea Rhizome, Rhizome of Atractylodes lancea De Candolle (Compositae)	3
Chorei	Polyporus Sclerotium, Sclerotia of Polyporus umbellatus Fries (Polyporaceae)	3
Bukuryou	Poria Sclerotium, Sclerotia of Wolfiporia cocos Ryvarden et Gilbertson (Polyporaceae)	3
Keihi	Cinnamon Bark, Bark of Cinnamomum cassia J. Presl (Lauraceae)	1.5

### 2.3 Immunofluorescence

Immunofluorescence was performed as described previously [11]. Briefly, PC-12 cells were cultured in glass-bottomed dishes (82-4945: Matsunami Glass Ind., Ltd., Osaka, Japan). Five days after LPS and Kampo treatment, the cells were fixed in 4% formaldehyde. Fixed cells were incubated for 30 min in a blocking solution (5% skim milk in phosphate-buffered saline; PBS with Tween-20; PBST). After washing with PBST, cells were incubated with primary antibodies (Anti-prohibitin 2; PHB2: GTX32812, GeneTex, Inc. Irvine, CA, USA; anti-GSK3β: 9315, Cell Signaling Technology, Danvers, MA, USA; anti-WNK: A301-514A, Bethyl Laboratories, Montgomery, TX, USA; anti-pSPAK (Ser373): 07-2273, Merck, KGaA, Darmstadt, Germany; anti-KCC2: AB3560P, Merck; anti- MECP2: ab2828, Abcam, Cambridge, UK; anti-REST: bs-2590R, Bios Antibodies Inc. Woburn, MA, USA). Secondary antibodies (Alexa Fluor 488 goat anti-rabbit IgG: A11008, Thermo Fisher Scientific, Waltham, MA, USA or Alexa Fluor 568 goat anti-rabbit IgG: A11011, Thermo Fisher Scientific) were used to visualize primary antibody binding. Primary antibodies were incubated at 4 °C overnight at a dilution of 1/1000 and secondary antibodies were incubated for 3 h at room temperature at a dilution of 1/500. Cells were washed five times with PBST at room temperature for 3 min each after each antibody incubation. To stain the nuclei, cells were incubated with 4’, 6-diamidino-2-phenylindole (DAPI: 0.5 μg/mL) at room temperature for 10 min. Fluorescent images were obtained using a BZ-8000 fluorescence microscope (Keyence Corporation, Osaka, Japan) from three separate dishes for each treatment. To measure the fluorescence intensity, ImageJ software (Rasband, W.S., ImageJ, U.S. The National Institutes of Health, Bethesda, Maryland, USA, http://rsb.info.nih.gov/ij/, 1997–2012) was used. The protein expression level was obtained by measuring the brightness of each cell in the field of view one by one, subtracting the average background brightness (average 3 places of brightness areas without cells in the field of view), and calculating the average. This method is only semiquantitative, but the results of fluorescent immunostaining and Western blotting have the same tendency [11].

### 2.4 Gene silencing with siRNA

PC-12 cells were transfected with synthetic siRNA corresponding to rat Phb2 (AM16708: Thermo Fisher Scientific) or Rage (sc-106985: Santa Cruz Biotechnology, Dallas, TX, USA) using Lipofectamine™ RNAiMAX Transfection Reagent (13778075: Thermo Fisher Scientific) according to the manufacturer’s protocol two days before LPS treatment. AccuTarget-Negative Control siRNA (SN-1003: Bioneer, Daejeon, Korea) was used as a control.

### 2.5 Measurement of mitochondrial ROS and membrane potential (Ψ_m_)

MitoSOX™ Red mitochondrial superoxide indicator (M36008: Thermo Fisher Scientific) was used to detect mtROS. To detect Ψ_m_, cells were treated with 5, 5′, 6, 6′ tetrachloro-1, 1′, 3, 3′-tetraethylbenzimidazolylcarbocyanine iodide (JC-1, T3168: Thermo Fisher Scientific) or rhodamine 123 (Rho 123, R302: Thermo Fisher Scientific) with slight modifications to the manufacturer’s protocol. Cells in glass-bottomed dishes were treated with 5 μM mitoSOX and 2 μM JC-1 or 10 μM Rho 123 in Hank’s balanced salt solution (HBSS; 084-08345: Fujifilm Wako) for 10 min, 30 min, or 20 min at 37 °C. After incubation, the medium was replaced with fresh HBSS. Fluorescent images were obtained using a BZ-8000 fluorescence microscope and ImageJ software was used to measure the fluorescence intensity.

### 2.6 Western blotting

Each cell lysate (20 μg per lane) was subjected to SDS-PAGE under reducing conditions. The proteins were subsequently blotted on a polyvinylidene difluoride (PVDF) membrane. After blocking with blocking solution (5% skim milk in PBST), the membranes were incubated with primary antibodies (GSK3β, WNK, pSPAK^ser373^, or KCC2 antibodies) in blocking solution at 4 °C overnight (dilution factor 1:1000). After washing five times with PBST, the membranes were incubated with peroxidase-conjugated anti-rabbit IgG antibody (#7074: Cell Signaling Technology; dilution factor 1:1000) at room temperature for 2 h. Immunoreactive proteins were visualized with ImmunoStar Zeta (Fujifilm Wako) using a ChemiDoc XRS Plus instrument (Bio-Rad Laboratories, Inc., Hercules, CA, USA). As a loading control, anti-β-actin antibody (4970L: Cell Signaling Technology) was used (dilution factor: 1:1000).

### 2.7 Quantitative polymerase chain reaction

Quantitative polymerase chain reaction (qPCR) was performed as described previously [27]. Briefly, total RNA was isolated from PC-12 cells using ISOGEN (311-02501: Nippon Gene, Toyama, Japan), and cDNA was prepared using ReverTra Ace (TRT-101: TOYOBO CO Ltd., Osaka, Japan). One ng of total RNA was used for the PCR reactions. The qPCR reactions were performed with an Applied Biosystems 7300 Real-Time PCR System using THUNDERBIRD® SYBR® qPCR mix (QPS-201: TOYOBO). The mRNA levels were normalized to β-actin mRNA levels. The PCR conditions were as follows: 40 cycles of amplification (95 °C 10 s, 60 °C 60 s) after one cycle of denaturation (95 °C, 10 min). Each experiment was performed in triplicate. Gene expression levels relative to β-actin were calculated using the _ΔΔ_Ct-method. Primer sequences were listed in Table 2.

**Table 2:** Primer sequences used in this study.

Primer Name	Primer sequence (5′-3′)
Oxt F	TGCCCCAGTCTTGCTTGCTGCCT
Oxt R	AGGGAAGACACTTGCGCATATCCAGGT
Rage F	AAACCTCTGATTCCTGATGGCAAAGG
Rage R	CAACCAACAGCTGAATGCCCTCT
β-actin F	CTAAGGCCAACCGTGAAAAG
β-actin R	TACATGGCTGGGGTGTTGA

### 2.8 Animal behavior test

Neonatal C57BL/6J mice raised by their parents (Japan SLC, Inc., Shizuoka, Japan) were used for the experiments. All animals were housed in an air-conditioned room at a temperature of 22 ± 2 °C under a 12 h light/dark cycle (lights on at 7:00 and off at 19:00) with *ad libitum* access to food (CE2; CLEA Japan, Tokyo, Japan) and water. On postnatal day one, pups were randomly assigned to the control group (Cont; n = 6), *P. g* LPS group (LPS; n = 8), *P. g* LPS + OXT group (+OXT; n = 4), *P. g* LPS + KSS group (+KSS; n = 4), or *P. g* LPS + KKT group (+KKT; n = 4). *P. g* LPS (10 mg/kg) was intraperitoneally administered to neonatal male mice with a Hamilton syringe. LPS was administered on the day of birth (3–8 h after the first breastfeeding was confirmed; single dose). Saline was administered to the control group. OXT (0.5 mg/kg), KSS (400 mg/kg), and KKT (100 mg/kg) were intraperitoneally administered 10 min before LPS treatment. The open-field test (OFT) and elevated plus maze test (EPM) were performed four weeks after birth. During the OFT, mice were placed in a 45 cm diameter field. The ratio of time around the center and near the wall over 5 min was recorded. For the EPM, mazes were constructed with four arms (10 cm × 45 cm each) and placed 45 cm above the floor. An open arm (without a wall) and a closed arm (with 25 cm walls) were placed perpendicular to each other. A mouse was placed in the center of each arm, and time in the open or closed arms over 5 min was monitored. Data were captured and analyzed using SMART v2.5 (Pamlab, Ltd, Barcelona, Spain). All animal procedures were conducted in strict accordance with the Principles of Laboratory Animal Care and Use and were approved by the Kagoshima University Animal Care and Use Committee guidelines (Approval number: D21009, D21012, D22013).

### 2.9 Statistical analysis

Student’s *t*-test was performed after the *F* test to compare two groups. One-way ANOVA with Scheffe’s *F* test was performed to compare three or more groups using Statcel4 software (OMS publishing, Saitama, Japan). *P* < 0.05 was considered statistically significant. The results are expressed as the means ± standard errors of the mean.

## 3 Results

### 3.1 Effect of *P. g* LPS on mitochondria in PC-12 cells

**Fig. 1:**
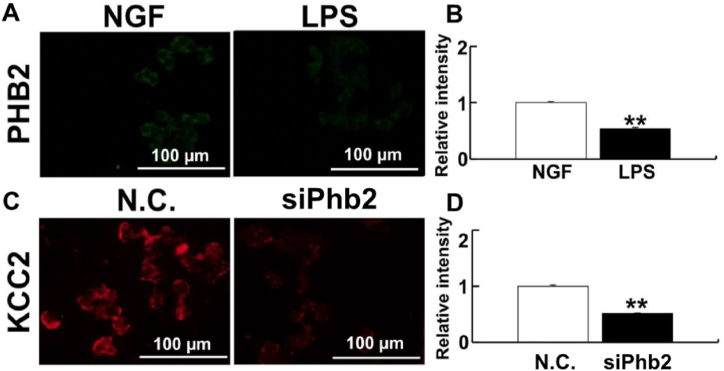
PHB2 regulation of KCC2 expression. (A) PHB2 expression in PC-12 cells detected by immunofluorescence after LPS treatment. (B) Relative intensity of PHB2. (C) Detection of KCC2 in PC-12 cells after *Phb2* knockdown. (D) Relative intensity of KCC2. LPS treatment decreases PHB2 and the knockdown of *Phb2* decreases KCC2 expression in PC-12 cells. Fluorescent images were obtained from three separate dishes for each treatment. **: *p* < 0.01 using student's *t*-test.

PHB2 plays an important role in maintaining mitochondrial membrane structure [28]. Therefore, we investigated changes in PHB2 expression levels after *P. g* LPS treatment in PC-12 cells. PHB2 expression decreased significantly compared to control in response to *P. g* LPS treatment (Fig. 1A and B). Knockdown of PHB2 by siRNA significantly decreased KCC2 expression, suggesting that mitochondrial dysfunction suppresses KCC2 expression (Fig. 1C and D). We confirmed the downregulation of *Phb2* gene expression after siPhb2 treatment (Supplemental Fig. 2). To evaluate mitochondria function, we measured Ψ_m_ and mtROS levels after *P. g* LPS treatment (Supplemental Fig. 3A). After *P. g* LPS treatment, mtROS levels increased significantly (Supplemental Fig. 3B) and Ψ_m_ decreased significantly, as detected by both JC-1 and Rh 123 (Supplemental Figs. 3C and D).

### 3.2 KSS and KKT protect mitochondrial dysfunction induced by *P. g* LPS

**Fig. 2:**
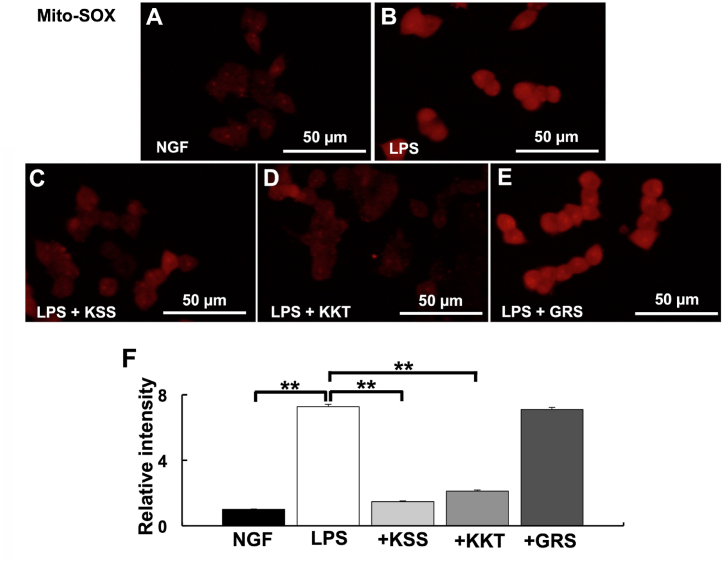
Detection of mtROS after *P. g* LPS and Kampo treatment. (A–E) Relative mtROS intensity was detected by mitoSOX. (A) NGF treatment (control). (B) *P. g* LPS treatment. (C) *P. g* LPS + Kamishoyosan treatment. (D) P. g LPS + Kamikihito treatment. (E) *P. g* LPS + Goreisan treatment. (F) Relative intensities of A–E. Relative mtROS were significantly increased after *P. g* LPS treatment. Pretreatment with Kamishoyosan or Kamikihito prevented the *P. g* LPS-induced changes in mtROS. Goreisan did not prevent the mtROS increase induced by *P. g* LPS. Fluorescent images were obtained from three separate dishes for each treatment. **: *p* < 0.01 vs. LPS by Scheffe’s F test.

**Fig. 3:**
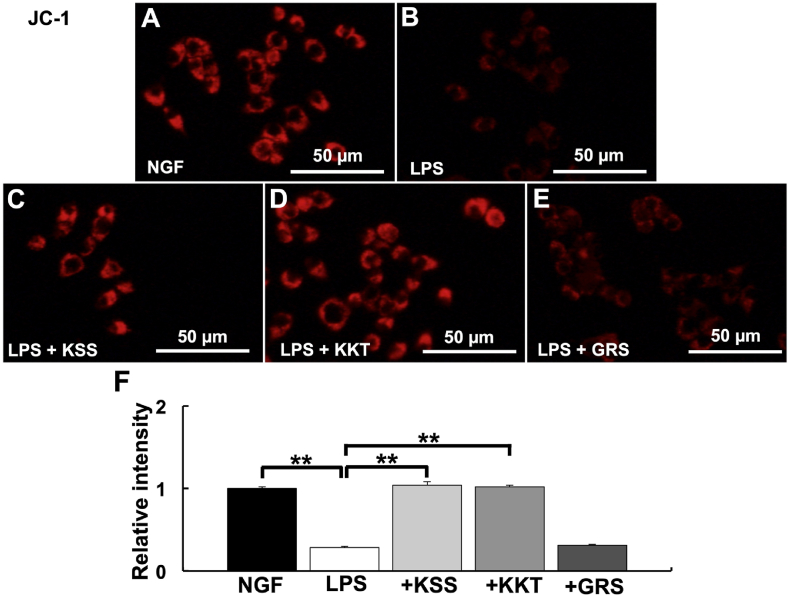
**Detection of mitochondrial membrane potential (Ψ**_**m**_**) after *P. g* LPS and Kampo treatment.** (A–E) Relative Ψ_m_ intensity was detected by JC-1. (A) NGF treatment (control). (B) *P. g* LPS treatment. (C) *P. g* LPS + Kamishoyosan treatment. (D) *P. g* LPS + Kamikihito treatment. (E) *P. g* LPS + Goreisan treatment. (F) Relative intensities of A-E. Relative Ψ_m_ was significantly decreased after *P. g* LPS treatment. Pretreatment with Kamishoyosan or Kamikihito attenuated *P. g* LPS-induced changes in Ψ_m_. Goreisan did not prevent the Ψ_m_ decrease induced by *P. g* LPS treatment. Fluorescent images were obtained from three separate dishes for each treatment. **: *p* < 0.01 vs. LPS by Scheffe’s F test.

The effects of KSS, KKT, and GRS on mitochondrial dysfunction induced by *P. g* LPS treatment were investigated using mitoSOX and JC-1. *P. g.* LPS treatment increased mtROS compared with the control (Fig. 2A and B). When cells were pretreated with KSS or KKT before *P. g* LPS treatment, mtROS did not increase compared with the control (Fig. 2C and D). In contrast, mtROS significantly increased compared with the control after pretreatment with GRS before *P. g* LPS treatment (Fig. 2E and F). Furthermore, Ψ_m_ did not decrease after pretreatment with KSS or KKT before *P. g* LPS treatment (Fig. 3A–D), but treatment with GRS and *P. g* LPS resulted in significantly decreased Ψ_m_ similar to LPS treatment alone (Fig. 3B, E, F).

### 3.3 GSK3β activation by LPS was suppressed by KSS or KKT treatment

**Fig. 4:**
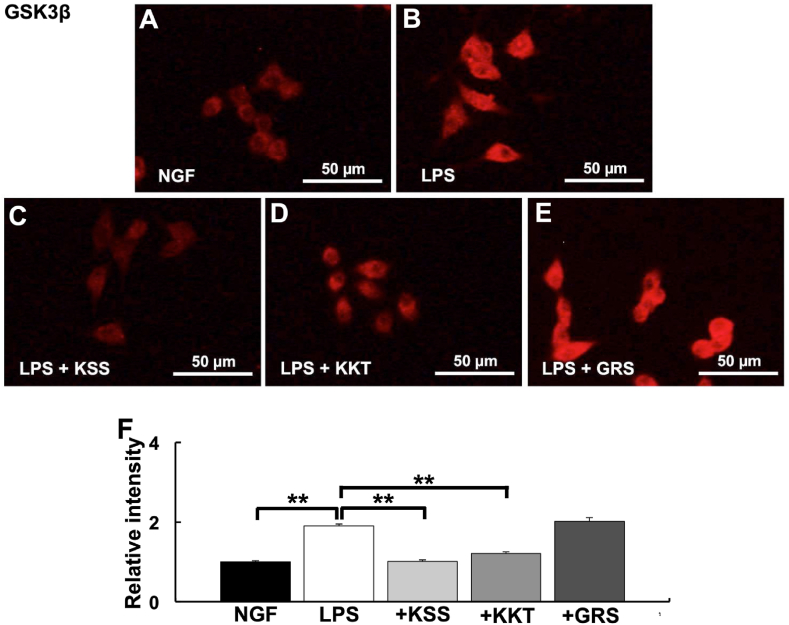
Expression of GSK3β after *P. g* LPS and Kampo treatment. (A–E) Relative GSK3β intensity was detected by immunostaining. (A) NGF treatment (control). (B) *P. g* LPS treatment. (C) *P. g* LPS + Kamishoyosan treatment. (D) *P. g* LPS + Kamikihito treatment. (E) *P. g* LPS + Goreisan treatment. (F) Relative intensities of A-E. Relative GSK3β expression were significantly increased after *P. g* LPS treatment. Pretreatment with Kamishoyosan or Kamikihito attenuated *P. g* LPS-induced changes in GSK3β expression. Goreisan did not prevent the GSK3β increase induced by *P. g* LPS treatment. Fluorescent images were obtained from three separate dishes for each treatment. **: *p* < 0.01 vs. LPS by Scheffe’s F test.

GSK3β is activated by *P. g* LPS treatment, leading to decreased KCC2 expression. Therefore, the effects of KSS, KKT, and GRS on GSK3β activation were analyzed. KSS and KKT prevented the increase in GSK3β expression induced by *P. g* LPS (Fig. 4A–D, F). In contrast, GRS treatment did not prevent the increase in GSK3β expression induced by LPS treatment (Fig. 4E and F).

### 3.4 Effects of LPS, KSS, and KKT treatment on the Wnk pathway

**Fig. 5:**
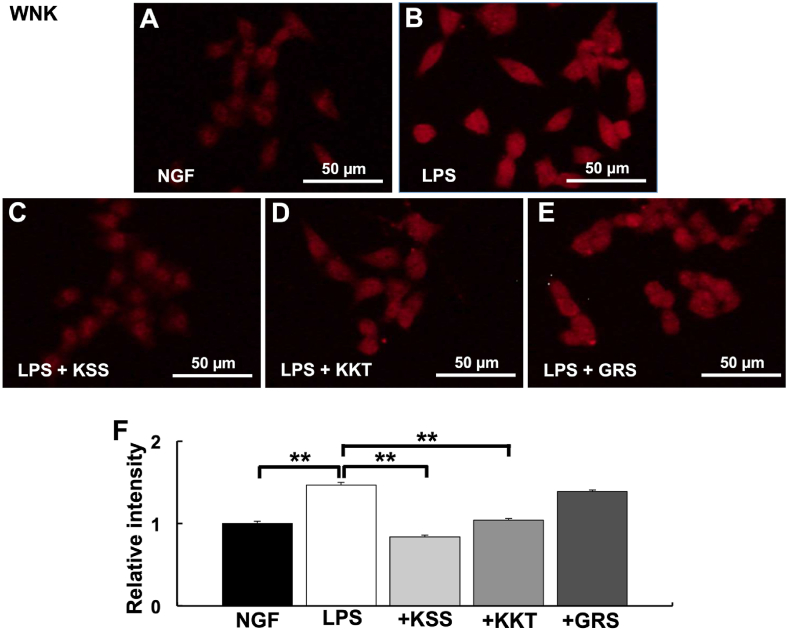
Expression of WNK after *P. g* LPS and Kampo treatment. (A–E) Relative WNK intensity was detected by immunostaining. (A) NGF treatment (control). (B) *P. g* LPS treatment. (C) *P. g* LPS + Kamishoyosan treatment. (D) *P. g* LPS + Kamikihito treatment. (E) *P. g* LPS + Goreisan treatment. (F) Relative intensities of A-E. Relative WNK expression was significantly increased after *P. g* LPS treatment. Pretreatment with Kamishoyosan or Kamikihito attenuated the *P. g* LPS-induced changes in WNK expression. Goreisan did not prevent WNK increases induced by *P. g* LPS treatment. Fluorescent images were obtained from three separate dishes for each treatment. **: *p* < 0.01 vs. LPS by Scheffe’s F test.

**Fig. 6:**
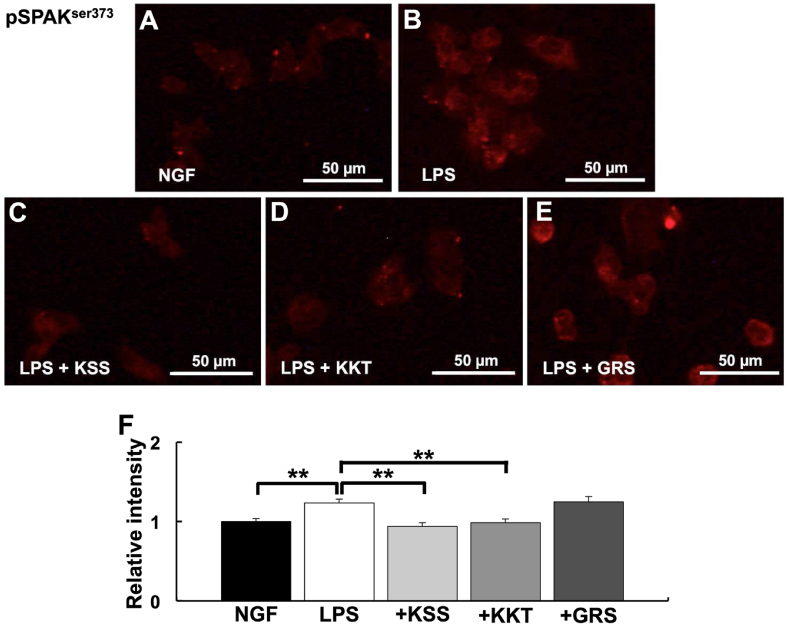
Expression of pSPAK^ser-373^ after *P. g* LPS and Kampo treatment. (A–E) Relative pSPAK^ser-373^ intensity detected by immunostaining. (A) NGF treatment (control). (B) *P. g* LPS treatment. (C) *P. g* LPS + Kamishoyosan treatment. (D) *P. g* LPS + Kamikihito treatment. (E) *P. g* LPS + Goreisan treatment. (F) Relative intensities of A-E. Relative pSPAK^ser-373^ expression was significantly increased after *P. g* LPS treatment. Kamishoyosan or Kamikihito pretreatment attenuated *P. g* LPS-induced changes in pSPAK^ser-373^ expression. Goreisan did not prevent the pSPAK^ser-373^ increase induced by *P. g* LPS treatment. Fluorescent images were obtained from three separate dishes for each treatment. **: *p* < 0.01 vs. LPS by Scheffe’s F test.

Because GSK3β acts as a positive effector downstream of the WNK-SPAK system, we next determined if the increase in GSK3β by *P. g* LPS treatment was mediated by the WNK-SPAK system. *P. g* LPS treatment increased WNK expression compared with NGF treatment (Fig. 5A and B). When cells were pretreated with KSS or KKT before *P. g* LPS treatment, the expression of WNK did not increase compared with the control (Fig. 5C and D). In contrast, the expression of WNK significantly increased compared with the control after pretreatment with GRS before *P. g* LPS treatment (Fig. 5E and F). As in the case of WNK, KSS and KKT, but not GRS, significantly suppressed the increases in pSPAK^ser373^ expression levels induced by *P. g* LPS treatment (Fig. 6A–F).

### 3.5 KSS and KKT prevent the decrease in KCC2 expression induced by *P. g* LPS

**Fig. 7:**
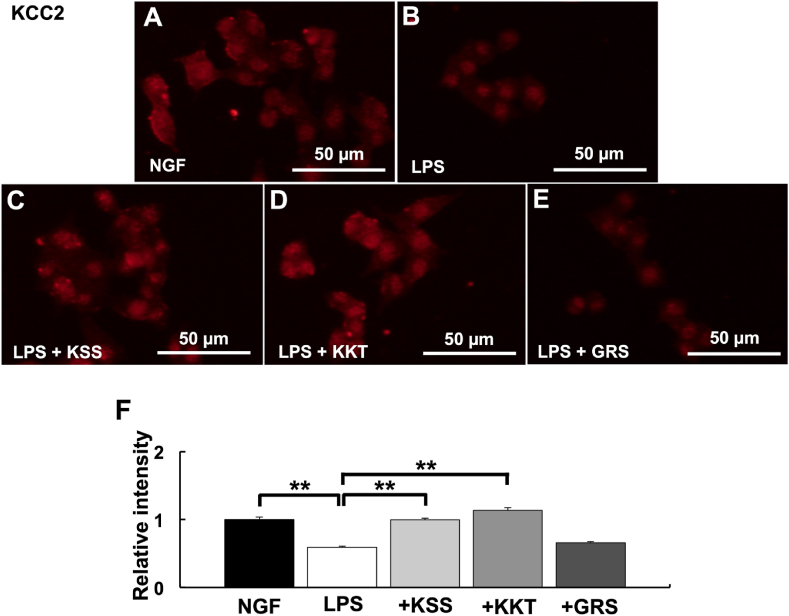
Expression of KCC2 after *P. g* LPS and Kampo treatment. (A–E) Relative KCC2 intensity detected by immunostaining. (A) NGF (control) treatment. (B) *P. g* LPS treatment. (C) *P. g* LPS + Kamishoyosan treatment. (D) *P. g* LPS + Kamikihito treatment. (E) *P. g* LPS + Goreisan treatment. (F) Relative intensities of A-E. Relative KCC2 expression was significantly decreased after *P. g* LPS treatment. Pretreatment with Kamishoyosan or Kamikihito attenuated the *P. g* LPS-induced changes in KCC2 expression. Goreisan did not prevent the KCC2 decrease induced by *P. g* LPS treatment. Fluorescent images were obtained from three separate dishes for each treatment. **: *p* < 0.01 vs. LPS by Scheffe’s F test.

**Fig. 8:**
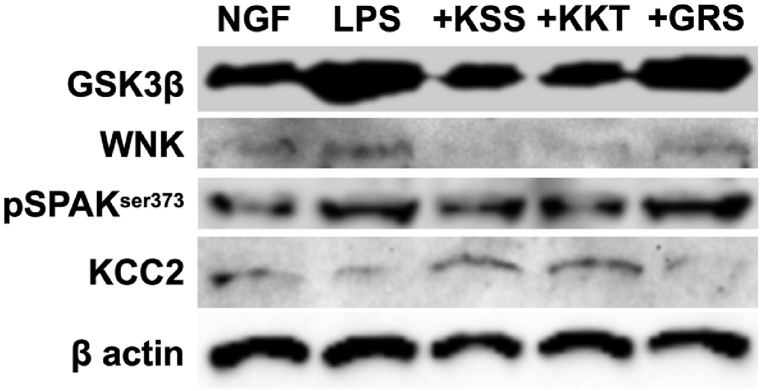
Western Blotting of GSK3β, WNK, pSPAK^ser373^, and KCC2 after *P. g* LPS and Kampo treatment. Twenty μg of each cell lysates were subjected to SDS-PAGE and blotted to PVDF membranes. LPS treatment up-regulated GSK3β, WNK, and pSPAK^ser-373^. KCC2 expression was down-regulated by LPS treatment. Pretreatment with Kamishoyosan or Kamikihito attenuated the *P. g* LPS-induced changes in these proteins. Goreisan did not prevent these protein expression changes induced by *P. g* LPS treatment. The full, non-adjusted images of Western blotting in this figure were shown in Supplemental Fig. 6.

*P. g* LPS downregulates KCC2 expression. Thus, we determined the effects of KSS or KKT treatment on decreased KCC2 expression induced by *P. g* LPS treatment. KSS and KKT pretreatment prevented the decreased KCC2 expression induced by *P. g* LPS (Fig. 7A–D, F). On the other hand, pretreatment with GRS did not prevent the decreased KCC2 expression induced by *P. g* LPS (Fig. 7E and F). To validate their consistency with immunostaining results western blotting of GSK3β, WNK, pSPAK^ser373^, and KCC2 after *P. g* LPS and Kampo treatment was performed. As a result, the same tendency was observed in Western blot and immunostaining (Fig. 8).

### 3.6 KSS and KKT prevent *P. g* LPS effects by different mechanisms

**Fig. 9:**
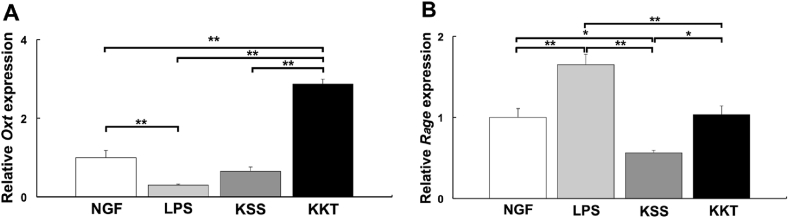
The relative gene expression changes of *oxytocin* (*Oxt*) and *Rage* after Kampo treatment by quantitative PCR (2h after treatment). NGF: control, LPS: *P. g* LPS, KSS; Kamishoyosan, KKT; Kamikihito. A: Kamikihito increased the expression of *Oxt*. *P. g* LPS treatment significantly decreased *Oxt* expression. In PC-12 cells, Kamikihito treatment significantly increased *Oxt* expression compared with the LPS treatment. B: Kamishoyosan decreased the expression of *Rage*. *P. g* LPS treatment significantly increased the expression of *Rage.* In PC-12 cells, KSS and KKT significantly decreased *Rage* expression compared with the LPS treatment. *: *p* < 0.05, **: *p* < 0.01 using Scheffe’s *F* test.

The effects of LPS, KSS, and KKT on *Oxt* and *Rage* gene expression levels were analyzed. The relative gene expression of *Oxt* was decreased significantly by LPS treatment and KKT treatment significantly increased *Oxt* expression than LPS treatment. KSS treatment did not significantly affect *Oxt* expression compared with LPS treatment (Fig. 9A). Thus, KKT, but not KSS, treatment increases the expression of *Oxt*. On the other hand, relative gene expression of *Rage* was increased significantly by LPS treatment, and KSS treatment significantly decreased *Rage* expression compared with LPS treatment. When KKT treatment was performed, *Rage* expression was significantly decreased compared with LPS treatment and comparable to NGF treatment (Fig. 9B).

### 3.7 Knockdown of *Rage* prevents nuclear translocation of REST and MECP2 after LPS treatment

**Fig. 10:**
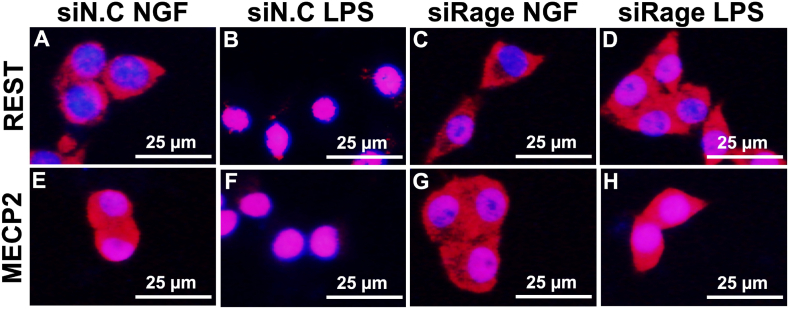
Nuclear translocation of REST and MECP2 after *P. g* LPS treatment is attenuated by siRage. The effects of *Rage* on the nuclear translocation of REST and MECP2 after LPS treatment were investigated. Immunostaining of REST (A–D), Immunostaining of MECP2 (E–H). LPS treatment induced the translocation of MECP2 and REST to the nucleus in PC-12 cells after transfection with the control si RNA (siN.C.) (A, B, E, F). Nuclear localization induced by LPS treatment was prevented when Rage was knocked down by siRNA treatment (C, D, G, H).

We investigated the effects of *Rage* on the nuclear translocation of REST and MECP2 induced by LPS treatment. Transfection with si*Rage* prevented the nuclear translocation of REST and MECP2 induced by *P. g* LPS treatment (Fig. 10A–H). Control siRNA did not affect the nuclear translocation of REST and MECP2 induced by LPS treatment (Fig. 10B, D, F, H). These results indicate that *Rage* is involved in *P. g* LPS signal transduction and the decrease in KCC2 expression.

### 3.8 KSS and KKT treatment prevent behavioral abnormalities caused by LPS treatment

**Fig. 11:**
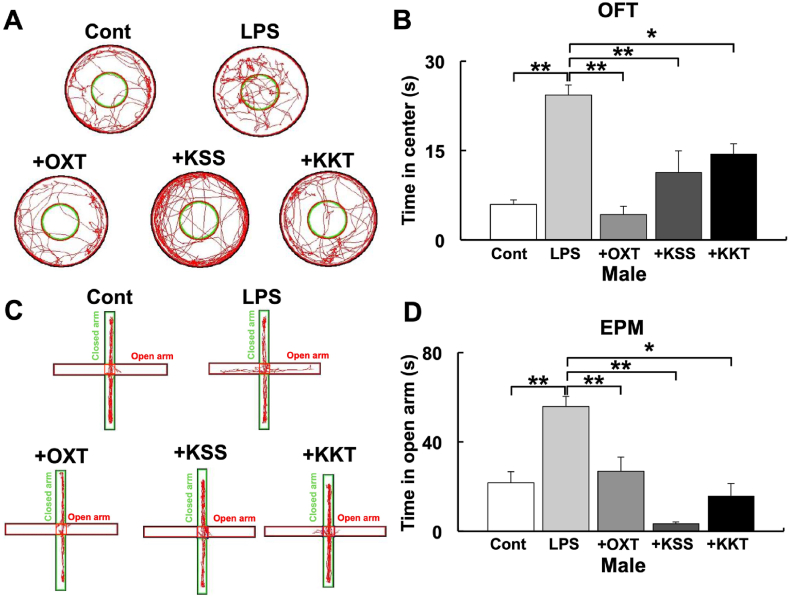
The influence of *P. g* LPS and Kampo on behavior tests in male mice. (A) Typical traces of mouse movement during the open-field test. Cont: saline, LPS: *P. g* LPS, +OXT; oxytocin + *P. g* LPS, + KSS; Kamishoyosan + *P. g* LPS, + KKT; Kamikihito + *P. g* LPS. (B) Time spent in the central compartment during the open-field test in each group. (C) Typical traces of mouse movement during the elevated plus maze test. (D) Time spent in the open arm during the elevated plus maze test. Mouse behavior was quantified and analyzed using SMART v2.5. *: *p* < 0.05, **: *p* < 0.01 using Scheffe’s *F* test (vs. *P. g* LPS).

Animal behavioral tests were performed to determine if KSS and KKT inhibit LPS effects in vivo. Intraperitoneal administration of *P. g* LPS in male mouse pups induced adolescent behavioral abnormalities, which were suppressed by KSS or KKT pretreatment, as evidenced by OFT (Fig. 11A and B) and EPM behavioral tests (Fig. 11C and D). On the other hand, KSS and KKT significantly attenuated *P. g* LPS effects on OFT but not EPM in female mice (Supplemental Figs. 4A and B).

## 4 Discussion

In this paper, we demonstrated that pretreatment with KSS and KKT can protect against mitochondrial dysfunction, upregulation of WNK-SPAK signaling, downregulation of KCC2 expression, and upregulation of GSK3β induced by *P. g* LPS treatment. We also demonstrated that KKT treatment increases *Oxt* expression and KSS treatment decreases *Rage* expression, resulting in decreased expression of *Tlr4*, the LPS receptor, and decreased nuclear translocation of MECP2 and REST, KCC2 modulators. Furthermore, KSS and KKT prevented *P. g* LPS-induced behavioral abnormalities in male mice. As far as we know, this is the first report demonstrating that KSS and KKT can prevent stress-induced KCC2 reduction and behavioral abnormalities.

PHB2 plays a protective role in many neurological defects, including neuroinflammation and cognitive function [29,30]. Our results show that the *P. g* LPS decreases KCC2 expression via the downregulation of PHB. The loss of KCC2 activity causes several neurological and psychiatric disorders, including epilepsy and schizophrenia [31,32]. Therefore, our results indicate that *P. g* LPS may cause psychiatric disorders via mitochondrial dysfunction.

LPS treatment affects mitochondrial function (e.g., fragmented morphology, decrease oxygen consumption ratio, Ψ_m_ decrease, and upregulation of ROS production) in glial cells [33]. LPS-induced IL-1β, NF-kb, and mtROS generation are suppressed by the superoxide scavenger mito-TEMPO in microglial cells [34]. Moreover, the LPS-induced inflammatory response is modulated by GSK3β [35,36], and GSK3β regulates mitochondrial activity [19]. We previously showed that OXT attenuates the suppression of KCC2 caused by *P. g* LPS treatment by inhibiting GSK3β signaling in PC-12 cells [11]. In this study, we showed that Kampo such as KSS and KKT suppress mtROS production, Ψ_m_ decrease, and the upregulation of GSK3β induced by *P. g* LPS treatment. These results suggest that mitochondrial dysfunction caused by *P. g* LPS treatment is regulated by GSK3β, and the dysfunction is attenuated by pretreatment with KSS and/or KKT. In contrast, GR*S* had no effect in this study. To date, there have been no reports that GRS is involved in the expression of oxytocin or RAGE. GRS exerts its action by regulating water content via aquaporin expression [37]; thus, although GRS has effects on the nervous system, GRS may not be involved in the mechanisms investigated in this paper, namely the LPS-induced mitochondrial dysfunction in PC-12 cells. The WNK-SPAK system regulates GSK3β [20], KCC2, and NKCC1 [21,22]. The WNK-SPAK system, which regulates blood pressure, is activated by the PI3K/AKT signaling pathway via inhibition of GSK3β phosphorylation at Ser-9 [38]. WNK and SPAK do not phosphorylate GSK3β directly, but may form trimmers and are involved in neurite elongation [20]. Neurite elongation is promoted by increased phosphorylation of Erk1/2 and Akt, which are promoted by KSS treatment in PC-12 cells [25]. Taken together with our results, the WNK-SPAK pathway may play an important role in neural maturation via the GSK3β pathway, including the activation of KCC2 expression, and WNK-SPAK pathway is inhibited by *P. g* LPS treatment. However, the inhibition of neural maturation by *P. g* LPS may be attenuated by Kampo treatment such as KSS.

Regarding the molecular mechanism of KSS and KKT, KSS increases the phosphorylation of Akt/Erk1/2 [25], and KKT increases the expression of OXT [4]. Phosphorylation of Akt leads to the inactivation of GSK3β via TLR-mediated phosphorylation at Ser-9 [39]. *P. g* LPS causes neuroinflammation [40], which may be mediated by GSK3β-dependent TLR4 signaling in mice [41]. We have previously shown that *P. g* LPS uses TLR4 as a receptor to induce *Il-1β* expression via GSK3β, translocate MECP2 and REST to the nucleus, and decrease KCC2, which is rescued by OXT pretreatment [11]. We did not demonstrate direct binding of KSS or KKT to TLR4 but showed that KSS suppresses the expression of RAGE, which also acts as a receptor for LPS and OXT (Fig. 9). Therefore, KSS decreases TLR4 signaling by decreasing RAGE expression and attenuates *P. g* LPS receptor-mediated downregulation of KCC2. This hypothesis is supported by the data showing that MECP2 and REST are not translocated to the nucleus in response to *P. g* LPS when Rage is knocked down with siRNA. OXT suppresses TLR4 in the rat spinal cord [42]. KKT enhances OXT expression and suppresses TLR4, leading to the suppression of *P. g* LPS signaling. Increased OXT expression induced by KKT may competitively inhibit *P. g* LPS binding to RAGE and suppress LPS signaling. Taken together, our results indicate that KSS and KKT inhibit *P. g* LPS signaling at the receptor level by different mechanisms.

**Fig. 12:**
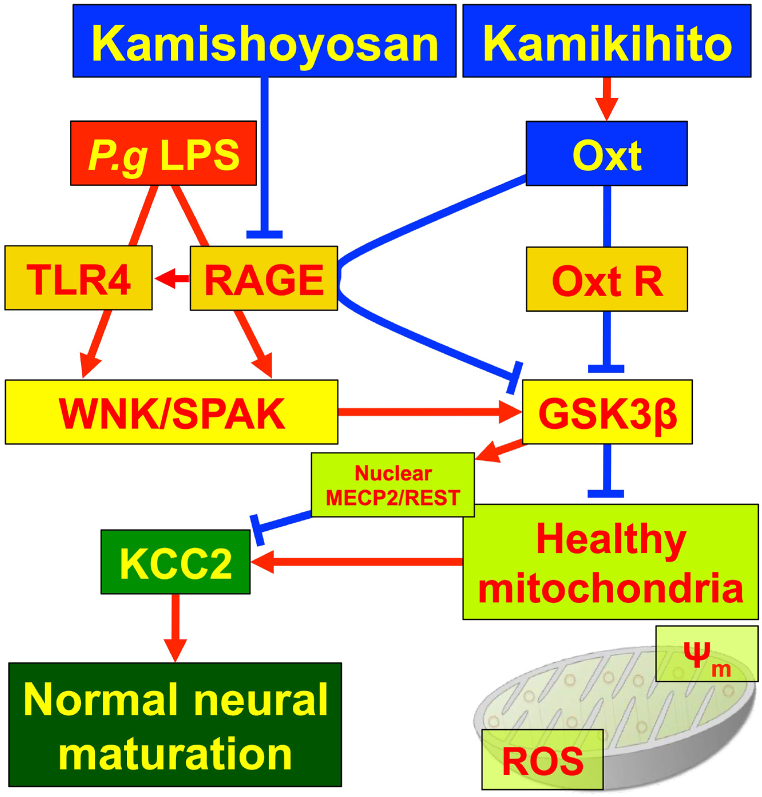
Schematic diagram of the signal cascade induced by *P. g* LPS treatment. *P. g* LPS reduces KCC2 expression. In this process, *P. g* LPS binds to TLR4 or RAGE. *P. g* LPS activates WNK/SPAK and WNK/SPAK activates GSK3β. GSK3β enhances the nuclear localization of REST and MECP2, which bind to the KCC2 promoter and downregulate KCC2 expression. GSK3β decreases mitochondrial membrane potential (Ψ_m_) and increases mitochondrial ROS (mtROS). Decreased Ψ_m_ and increased mtROS inactivate KCC2 expression. Decreased KCC2 expression inhibits normal neural maturation. *Kmishoyosan* decreases the expression of *Rage,* which decreases *P. g* LPS receptor *Tlr4* expression (Supplemental Fig. 5). *Kamikihito* increases the expression of oxytocin (Oxt), which inactivates GSK3β and leads to an increase in KCC2 expression.

The effects of *P. g* LPS in animal behavioral experiments are presumed to be caused by anxiolytic effects and reduced attention, similar to maternal separation experiments [43]. Furukawa et al. showed that maternal separation reduced anxiety, attention, and KCC2 expression and increased aggression in mouse pups compared with no maternal separation. Increased aggressive behavior is a common feature of many psychiatric disorders [44]. Aggression also increases after stress, such as social stress, and is suppressed by KSS administration after stress [3]. In this study, pre-administration of KSS and KKT rescued LPS-induced behavioral abnormalities, suggesting that KSS and KKT are effective against stress-induced nervous system abnormalities. Our results show a significant difference in males, but significant effects were not detected in the EPM test in females (Supplemental Fig. 4B). It has been reported that EPM is more susceptible to external influences such as lightning levels than OFT [45]. In addition to this, the sex difference of the EPM may have been caused by the differences in hormones and neurotransmitters such as estrogen and OXT. The behavior of female mice in the EPM test changes with the amount of estrogen [46]. We found that OXT expression was significantly higher in female mice compared with the expression in male mice (Supplemental Figs. 4C and D). However, the amount of OXT alone cannot explain the results of this behavioral change but changes in estrogen levels during the sexual cycle may affect behavior. The expression of 5-HT may also be different in males and females [3]. OXT releases 5-HT in the nucleus accumbens to support social reward [47], and KSS treatment increases 5-HT expression [3]. These hormones and neurotransmitters may influence behavior. OXT treatment through the nasal mucosa has been effective in the treatment of patients with autism [7,8]. Nasal administration of OXT after irradiation also rescued KCC2 expression, which was decreased by γ-irradiation in the hippocampal region of mice [48]. Intracellular Cl^−^ ion concentration decreases in response to X-ray irradiation in mouse primary cultures were attenuated by OXT treatment [48]. These results suggest that KSS and KKT may be effective against neurological disorders even when they are administered after the onset of the disease. Further investigations are needed, including determining the appropriate concentration for therapeutic effects and determining the long-term (repeated) effects of treatment. The results of this study (summarized in Fig. 12) indicate that KSS and KKT may be viable options for the treatment of psychiatric disorders mediated by decreased KCC2 and our results may contribute to the development of causative therapies.

## Ethical statement

(a) The name of the Ethics Committee: Principles of Laboratory Animal Care and Use and were approved by the Kagoshima University Animal Care and Use Committee guidelines. (b) The date of this approval; and (c) the number of the document: 06/07/2021; D21009, 06/12/2021; D21012, 11/29/2022; D22013.

## Sources of funding

There are no funders to report for this submission.

## Data availability statement

The data underlying this article are available in the article and in its online supplementary material.

## CRediT authorship contribution statement

**Kazuo Tomita:** Writing – review & editing, Writing – original draft, Validation, Methodology, Investigation, Formal analysis, Conceptualization, All authors have read and approved the final manuscript. **Yukiko Oohara:** Writing – original draft, Validation, Investigation, Formal analysis. **Kento Igarashi:** Writing – original draft, Validation, Investigation, Formal analysis. **Junichi Kitanaka:** Writing – review & editing, Validation, Conceptualization. **Nobue Kitanaka:** Writing – review & editing, Validation, Conceptualization. **Koh-ichi Tanaka:** Writing – review & editing, Conceptualization. **Mehryar Habibi Roudkenar:** Writing – review & editing, Conceptualization. **Amaneh Mohammadi Roushandeh:** Writing – review & editing, Conceptualization. **Mitsutaka Sugimura:** Writing – review & editing, Resources, Conceptualization. **Tomoaki Sato:** Writing – review & editing, Validation, Supervision, Resources, Project administration, Conceptualization.

## Declaration of competing interest

The authors declare that they have no known competing financial interests or personal relationships that could have appeared to influence the work reported in this paper.

## Abbreviations

KSS: *Kamishoyosan*
KKT: *Kamikihito*
5-HT: 5-hydroxytryptamine
OXT: Oxytocin
GSK3β: glycogen synthase kinase 3β
KCC2: K^+^-Cl^-^ co-transporter 2
LPS: lipopolysaccharide
*P. g*: *Porphyromonas gingivalis*
TLR: toll-like receptor
IL-1β: interleukin-1 beta
REST: RE1-silencing transcription factor
MECP2: methyl CpG binding protein 2
Ψ_m_: mitochondrial membrane potential
mtROS: mitochondrial reactive oxygen species
WNK-SPAK: with-no-lysine kinase- SPS1-related proline/alanine-rich kinase
NKCC1: Na^+^-K^+^-Cl^-^ co-transporter 1
RAGE: receptor for advanced glycation end products
HS: horse serum
PS: penicillin-streptomycin
NGF: nerve growth factor
GRS: *Goreisan*
PBS: phosphate-buffered saline
PBST: PBS with Tween-20
PHB: prohibitin
DAPI: 4′,6-diamidino-2-phenylindole
JC-1: 5, 5′, 6, 6′ tetrachloro-1, 1′, 3, 3′-tetraethylbenzimidazolylcarbocyanine iodide
Rho 123: rhodamine 123
HBSS: Hank’s balanced salt solution
qPCR: quantitative polymerase chain reaction
OFT: open-field test
EPM: elevated plus maze test
