# Supplementary material for: Kamishoyosan and Kamikihito protect against decreased KCC2 expression induced by the P. gingivalis lipopolysaccharide treatment in PC-12 cells and improve behavioral abnormalities in male mice
Source: Heliyon. 2023 Nov 25;9(12):e22784. doi: 10.1016/j.heliyon.2023.e22784 (PMC10711140; doi:10.1016/j.heliyon.2023.e22784)
Supplement: Multimedia component 1 [file mmc1.pptx]

## Slide 1
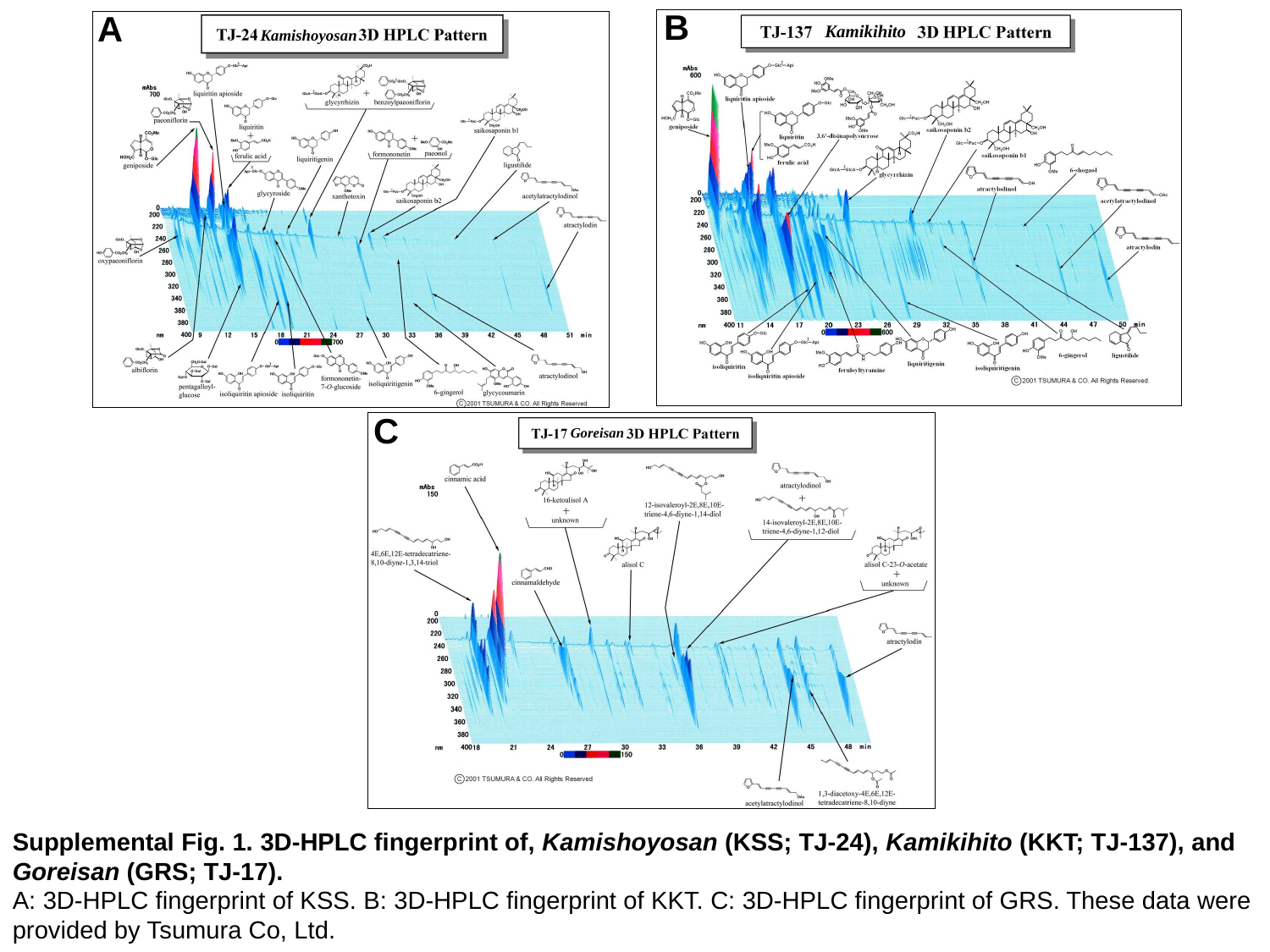

A
 B
Kamikihito
Kamishoyosan
C
Goreisan
Supplemental Fig. 1. 3D-HPLC fingerprint of, Kamishoyosan (KSS; TJ-24), Kamikihito (KKT; TJ-137), and Goreisan (GRS; TJ-17).
A: 3D-HPLC fingerprint of KSS. B: 3D-HPLC fingerprint of KKT. C: 3D-HPLC fingerprint of GRS. These data were provided by Tsumura Co, Ltd.

## Slide 2
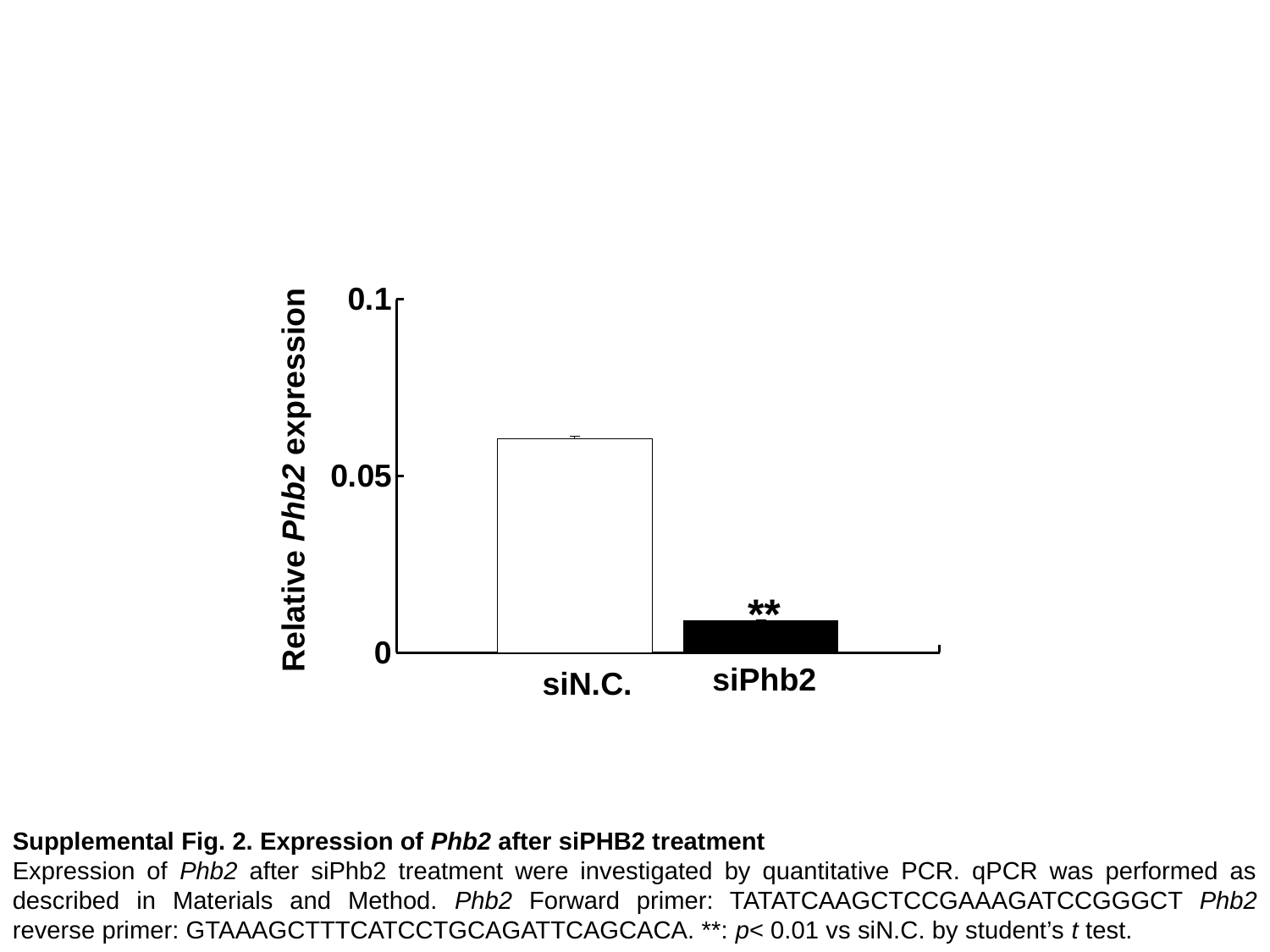

### Chart
| Category | N.C. | siPHB2 |
|---|---|---|
| PHB2 | 0.060624011902217806 | 0.009077796227351026 |Relative Phb2 expression
**
siPhb2
siN.C.
Supplemental Fig. 2. Expression of Phb2 after siPHB2 treatment
Expression of Phb2 after siPhb2 treatment were investigated by quantitative PCR. qPCR was performed as described in Materials and Method. Phb2 Forward primer: TATATCAAGCTCCGAAAGATCCGGGCT Phb2 reverse primer: GTAAAGCTTTCATCCTGCAGATTCAGCACA. **: p< 0.01 vs siN.C. by student’s t test.

## Slide 3
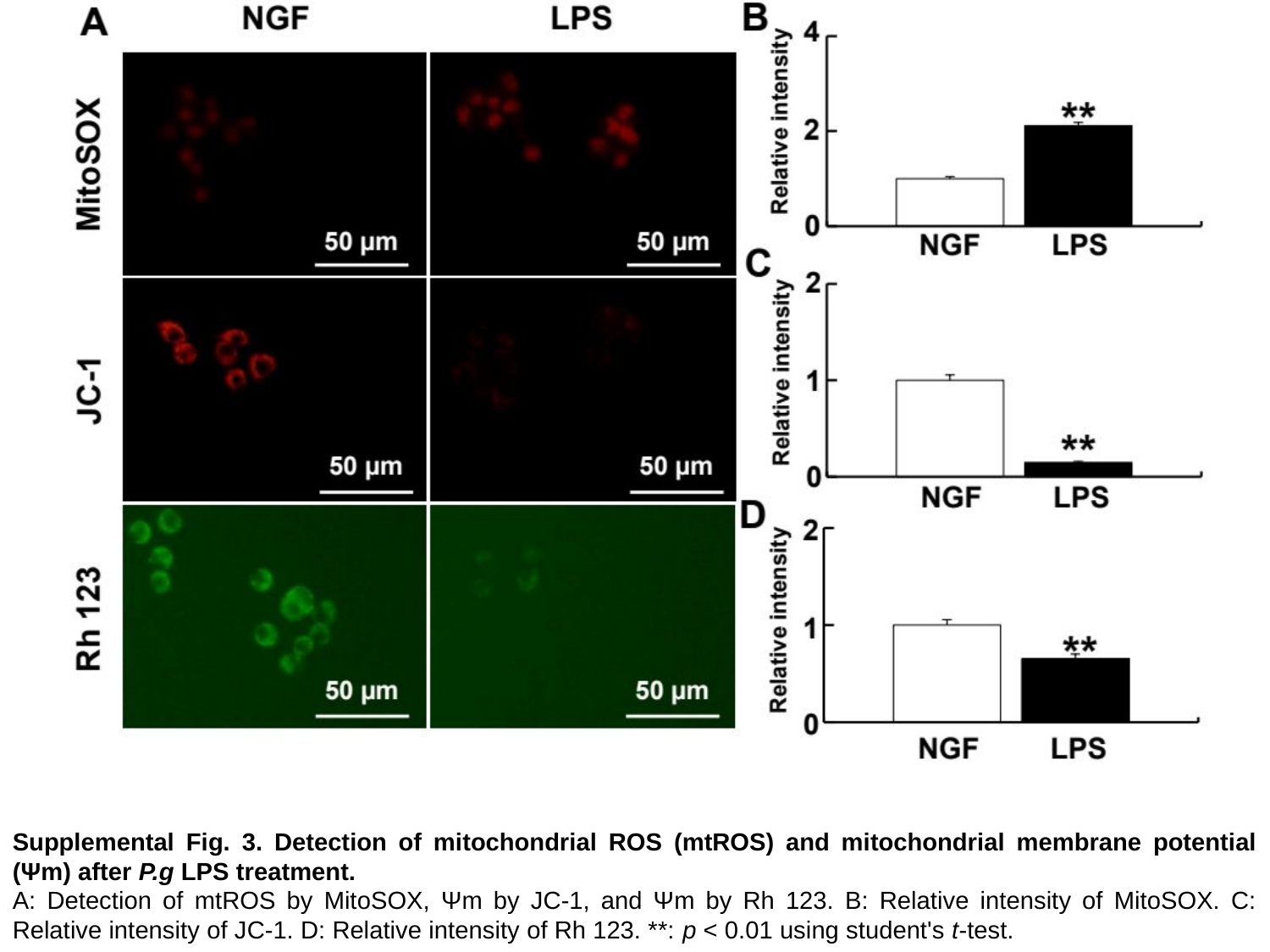

Supplemental Fig. 3. Detection of mitochondrial ROS (mtROS) and mitochondrial membrane potential (Ψm) after P.g LPS treatment.
A: Detection of mtROS by MitoSOX, Ψm by JC-1, and Ψm by Rh 123. B: Relative intensity of MitoSOX. C: Relative intensity of JC-1. D: Relative intensity of Rh 123. **: p < 0.01 using student's t-test.

## Slide 4
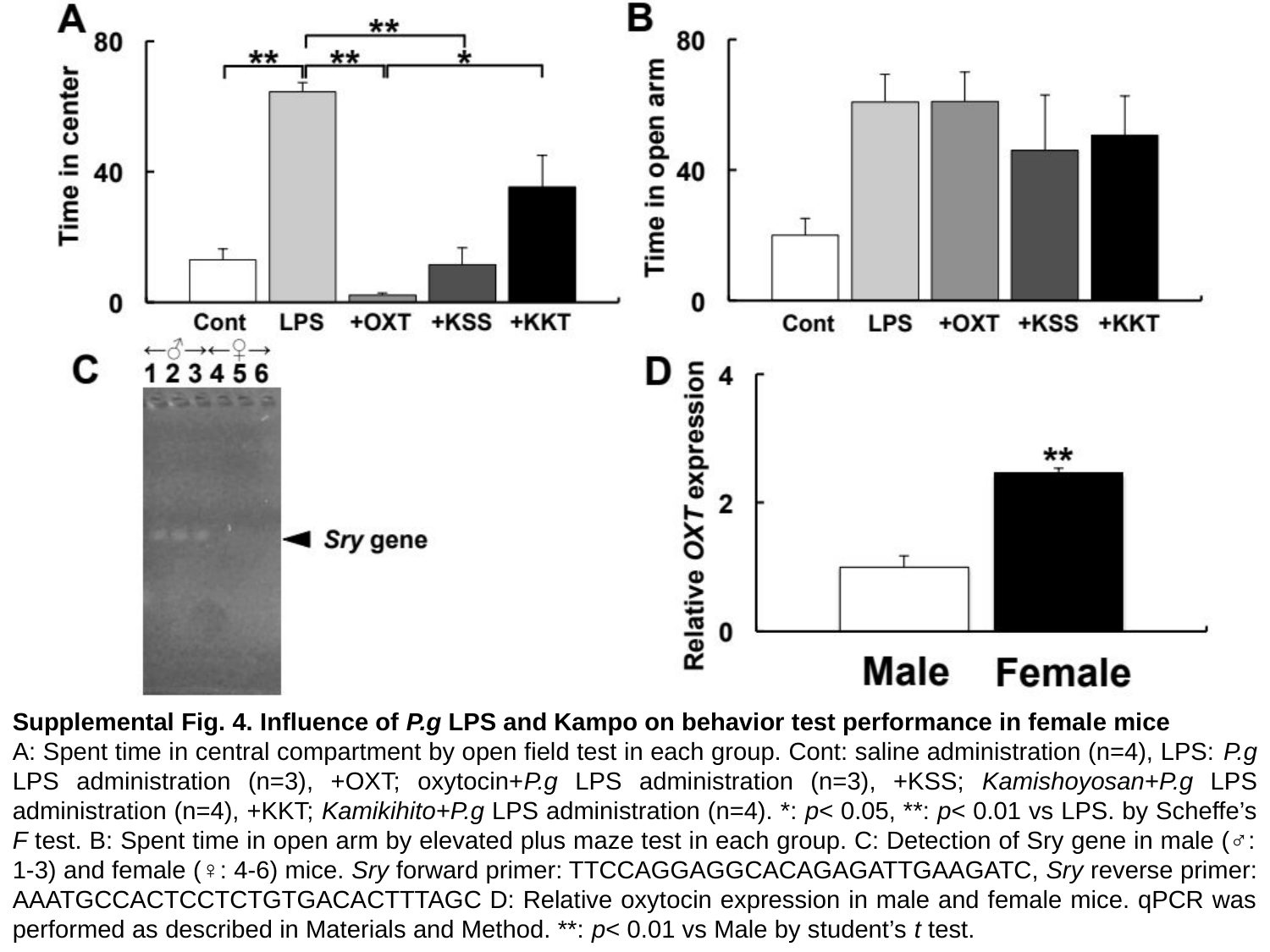

Supplemental Fig. 4. Influence of P.g LPS and Kampo on behavior test performance in female mice
A: Spent time in central compartment by open field test in each group. Cont: saline administration (n=4), LPS: P.g LPS administration (n=3), +OXT; oxytocin+P.g LPS administration (n=3), +KSS; Kamishoyosan+P.g LPS administration (n=4), +KKT; Kamikihito+P.g LPS administration (n=4). *: p< 0.05, **: p< 0.01 vs LPS. by Scheffe’s F test. B: Spent time in open arm by elevated plus maze test in each group. C: Detection of Sry gene in male (♂: 1-3) and female (♀: 4-6) mice. Sry forward primer: TTCCAGGAGGCACAGAGATTGAAGATC, Sry reverse primer: AAATGCCACTCCTCTGTGACACTTTAGC D: Relative oxytocin expression in male and female mice. qPCR was performed as described in Materials and Method. **: p< 0.01 vs Male by student’s t test.

## Slide 5
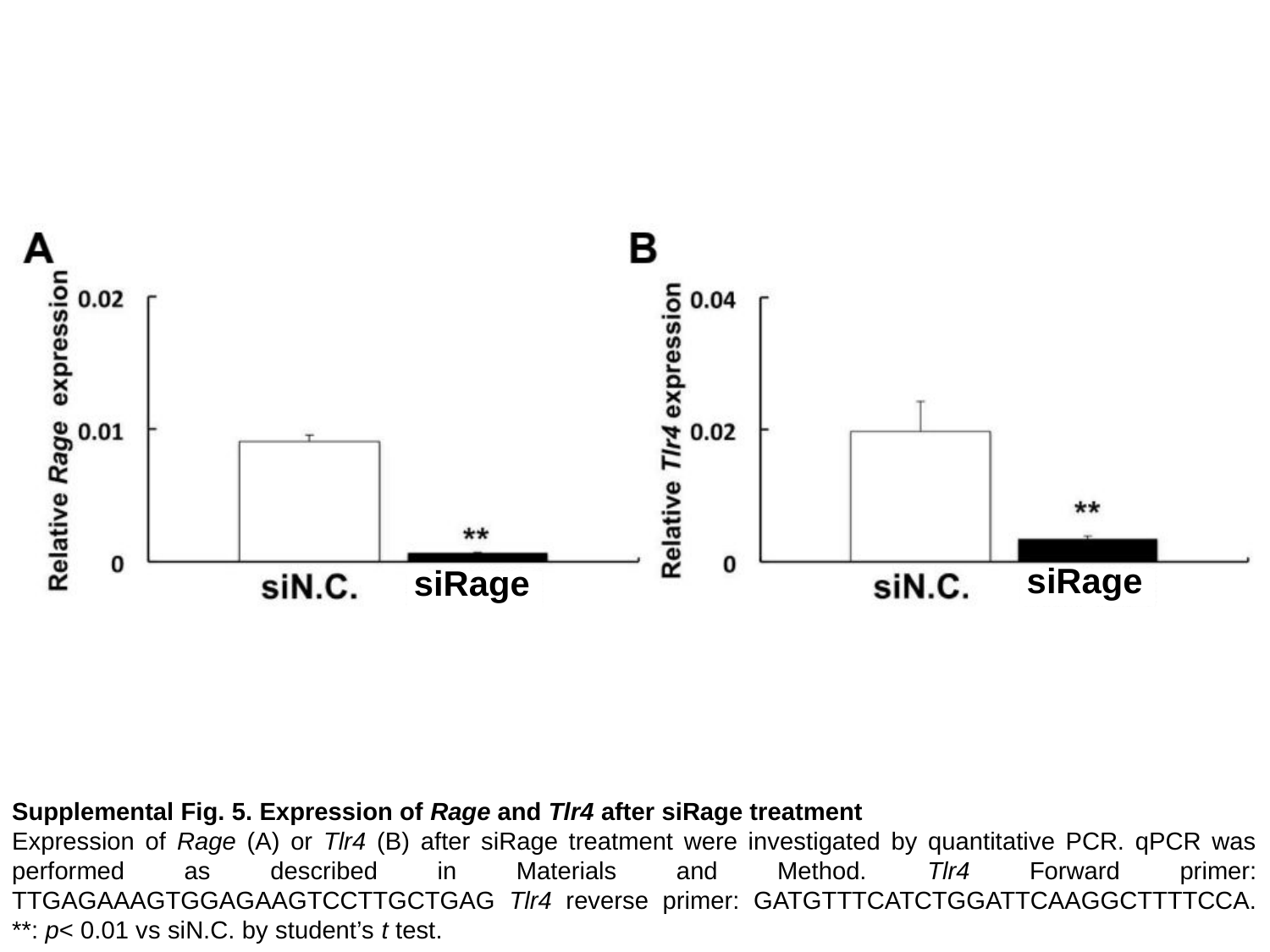

siRage
siRage
Supplemental Fig. 5. Expression of Rage and Tlr4 after siRage treatment
Expression of Rage (A) or Tlr4 (B) after siRage treatment were investigated by quantitative PCR. qPCR was performed as described in Materials and Method. Tlr4 Forward primer: TTGAGAAAGTGGAGAAGTCCTTGCTGAG Tlr4 reverse primer: GATGTTTCATCTGGATTCAAGGCTTTTCCA. **: p< 0.01 vs siN.C. by student’s t test.

## Slide 6
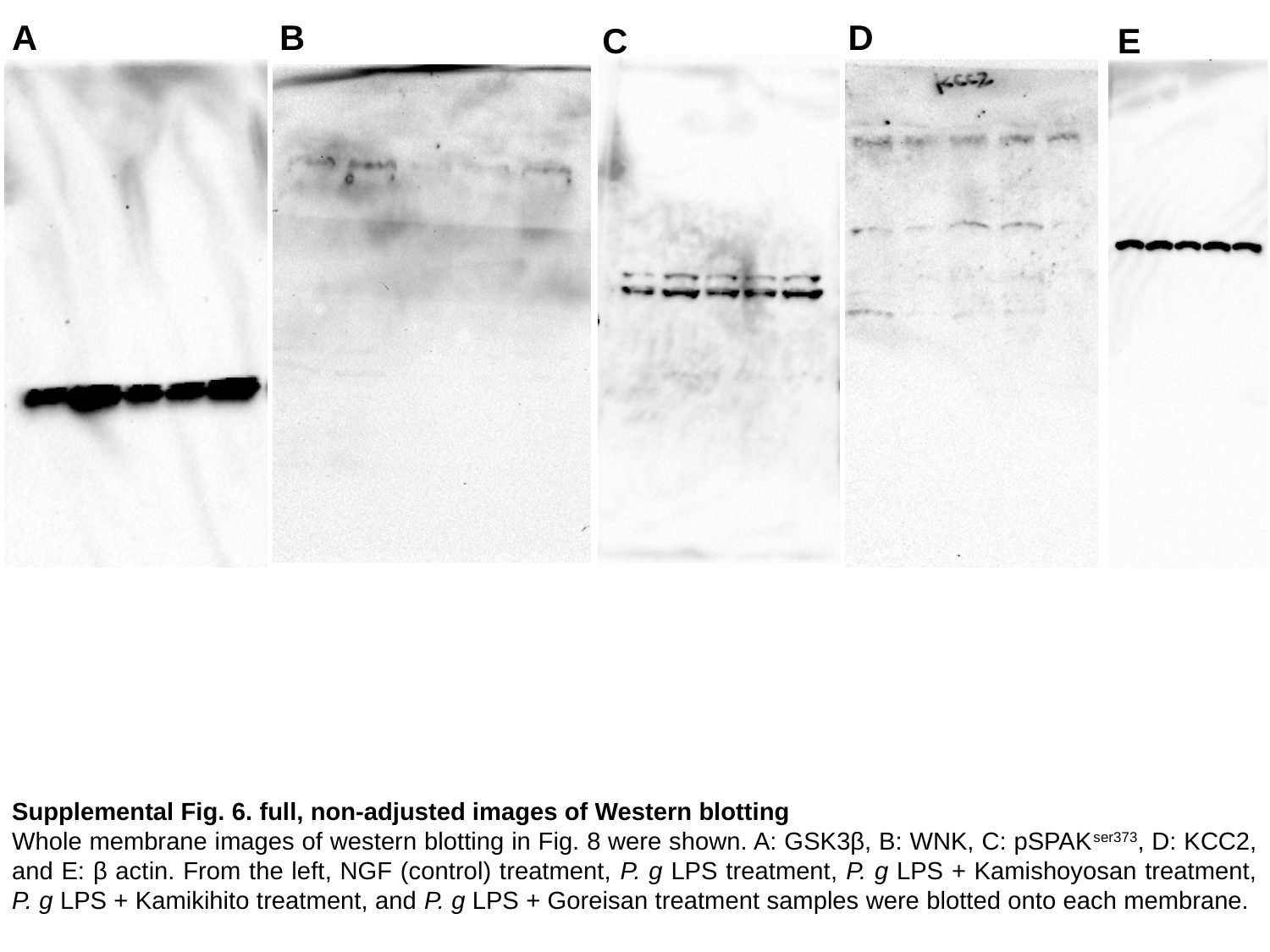

B
 D
A
C
E
Supplemental Fig. 6. full, non-adjusted images of Western blotting
Whole membrane images of western blotting in Fig. 8 were shown. A: GSK3β, B: WNK, C: pSPAKser373, D: KCC2, and E: β actin. From the left, NGF (control) treatment, P. g LPS treatment, P. g LPS + Kamishoyosan treatment, P. g LPS + Kamikihito treatment, and P. g LPS + Goreisan treatment samples were blotted onto each membrane.
